# Factors Affecting Age at Initial Autism Spectrum Disorder Diagnosis in a National Survey

**DOI:** 10.1155/2011/874619

**Published:** 2011-07-25

**Authors:** Rebecca E. Rosenberg, Rebecca Landa, J. Kiely Law, Elizabeth A. Stuart, Paul A. Law

**Affiliations:** ^1^Department of Medical Informatics, Kennedy Krieger Institute, Baltimore, MD 21211, USA; ^2^Center for Autism and Related Disorders, Kennedy Krieger Institute, Baltimore, MD 21211, USA; ^3^Department of Psychiatry, Johns Hopkins University School of Medicine, Baltimore, MD 21205, USA; ^4^Department of Pediatrics, Johns Hopkins University School of Medicine, Baltimore, MD 21205, USA; ^5^Department of Mental Health, Johns Hopkins Bloomberg School of Public Health, Baltimore, MD 21205, USA

## Abstract

Entry into early intervention depends on both age of first parent concern (AOC) and age at initial autism spectrum disorder (ASD) diagnosis (AOD). Using data collected from a national online registry from 6214 children diagnosed with an ASD between 1994 and 2010 in the US, we analyzed the effect of individual, family, and geographic covariates on AOC and AOD in a multivariate linear regression model with random effects. Overall, no single modifiable factor associated with AOC or AOD emerged but cumulative variation in certain individual- and family-based features, as well as some geographic factors, all contribute to AOC and AOD variation. A multipronged strategy is needed for targeted education and awareness campaigns to maximize outcomes and decrease disparities in ASD care.

## 1. Introduction

The diagnosis of autism spectrum disorders (ASDs), a group of neuropsychiatric disorders characterized by social and communication deficits and repetitive behaviors, has become increasingly common [1], affecting more than one in 110 children in the US [2]. Current subtypes in the American Psychiatric Association's Diagnostic and Statistical Manual of Mental Disorders (text revision) (DSM IV-TR) [3] include autistic disorder (AD), pervasive developmental disorder-not otherwise specified (PDD-NOS), and Asperger disorder (colloquially, Asperger syndrome; AS) and are referred to collectively in this paper, along with other community diagnoses such as “PDD” and “ASD”, as the autism spectrum disorders (ASDs). 

Lifetime costs for an individual with ASD can exceed $1.2 million [4, 5], and there is no known prevention or cure. Because early intervention has been shown to improve levels of functioning and rate of development, prompt access to effective [6, 7] early intervention services may ultimately permit less restrictive and less expensive educational placements. The gains in child functioning made possible through early intervention may also [8] be associated with reduced family and community burden [9–12]. 

Timely diagnosis is critical for enrollment in intervention services as early in life as possible, in order to capitalize on neuroplasticity [13]. Although diagnosis of ASD is possible in children as young as 14 months of age [13], most children with autism are not diagnosed until after three years of age [14, 15]. Understanding factors associated with delay in diagnosis, including both timing of age of parent concern (AOC) and age of initial diagnosis (AOD), may inform public awareness campaigns, screening guidelines, and professional education programming with the aim of lowering the age at which autism is detected. This would result in earlier access to intervention for children with autism. 

Age of parent concern about developmental delays or atypical patterns may be as early as birth for some children [16], but average AOC in ASD occurs around 12 to 18 months [16–22] and typically involves social and communication skills [16, 17, 23, 24]. Variation in AOC may reflect true differences in developmental trajectories of ASD subtypes or underlying causes [25–28], but there is limited or conflicting research on the effects of gender, family, community, and secular factors on AOC [25, 26, 29–31]. 

AOD has been more closely examined in the literature. Estimates of average AOD in ASD vary, ranging from 3.9 to 5.7 years depending on research design [14, 32–38]. Certain characteristics have consistently emerged as factors affecting AOD, including specific diagnosis; age of diagnosis for AD (3.1 to 5.5 years) or PDD-NOS (3.9 to 4.2 years) is much younger than average age of AS diagnosis (7 to 9 years) [14, 25, 34, 38, 39]. At the family level, higher parent education and affluence are associated with earlier AOD and increased likelihood of diagnosis [38, 40–43]. Local and systems factors, such as increased rurality and lower community affluence, have been associated with delays in AOD [33, 40–42, 44, 45].

Identification of significant and modifiable factors affecting AOC and AOD is critical to effective ASD-specific early intervention that maximizes outcomes. The previous literature on AOC and AOD has not integrated, into one study, both AOC and AOD, child factors (such as intellectual functioning, birth order, history of regression, initial ASD diagnosis), family context factors (such as maternal education, race/ethnicity), and geographical factors (such as region and county income). Further, while the largest epidemiologic [33] and administrative database [14] studies of AOD have examined multiple factors associated with AOD, neither had examined the AOC in the same population. As well, factors such as history of autistic regression and intellectual disability and family socioeconomic status were not included. 

Therefore, in an effort to identify modifiable opportunities for optimizing early intervention, our goals in this study were to identify and confirm parent-reported individual, family and geographic factors influencing AOC and AOD using the largest ASD dataset to date, an online open national registry, the Interactive Autism Network (IAN), and a registry with validated [46] and verified [47] parent-reported data. We hypothesized, based on the literature, that AOD but not AOC is affected by individual factors such as race, ethnicity, diagnosis, and gender, and family factors like maternal education, as well as by systems factors such as region and county income [25, 26, 29–31]. Identifying such variability could then help clinicians, families, and policy-makers focus on interventions to reduce disparities in ASD care.

## 2. Method

### 2.1. Participants

To investigate factors associated with variation in ages of first concern and diagnosis, we used the online Interactive Autism Network (IAN) database. IAN is an online US-based research database begun in April 2007 [48], with more than 33,000 individuals enrolled as of July 2010, including more than 13,000 affected individuals with professionally diagnosed ASD and their immediate family members (http://www.ianproject.org/). Families are recruited through a variety of mechanisms, including online media, provider referral, and support groups. All data are submitted by families who electronically consent, under the auspices of the Johns Hopkins Medicine Institutional Review Board (#NA_00002750). The database is continually updated and recruitment is ongoing. 

The current analysis was conducted with data extracted on July 1, 2010, from all subjects aged 0 to 21 years at the time of primary history questionnaire completion (*n* = 7871) with a diagnosis other than childhood disintegrative disorder; some evaluators use non-DSM-IV-TR labels such as “ASD” or “PDD”, referred to in this paper as “Other ASD”. Participants were excluded if our database did not contain information on maternal education (*n* = 428), date of initial diagnosis (month and year), inconsistent or missing date of diagnosis (*n* = 1073) or missing initial diagnosis (*n* = 14), or diagnosed before 1994 (*n* = 48), since the overall *n* for those years was <30 each year. We excluded all data from participants whose parents did not express concern prior to the time of diagnosis or did not provide any information on AOC (*n* = 25), who reported AOC that conflicted with AOD (*n* = 57) outside the range for estimation error or due to recall (e.g., AOC = 3 years and AOD = 1.5 years) or due to concerns of overall reliability, leaving a total of 6214 participants.

### 2.2. Measures

The IAN Project data collection consists of multiple topic-specific forms, authored by the IAN Research team in collaboration with other researchers. Questionnaires are available at http://www.iancommunity.org/cs/ian_research_questions/ian_research_questions. All families complete the initial registration and then are invited to complete several other questionnaires including a profile on each affected child. These surveys were developed by IAN staff in collaboration with members of the IAN Science Advisory Committee, piloted with families, and revised as needed.

 Our dependent variables were AOC and AOD. For AOC, parents were asked, “At what age did you first have concerns about your child's development?”. Parents could report open-ended AOC and/or 6-month-interval categories. For ease of presentation of AOC, the midpoint of each category was used when only approximate AOC interval was reported; for example, if concern was “Birth-6 months”, then 3 months was the AOC. This pattern was used for all participants missing specific age at concern (*n* = 248), thereby equally distributing any biases. 

For AOD, parents were asked specific date of initial ASD diagnosis. AOD was calculated by comparing date of birth and date of diagnosis. If parents gave only the month and year of diagnosis, then day of diagnosis was imputed as the midpoint (15th) of the month to enable consistent calculation of AOD. All participants included at least month/year for AOD.

Independent variables extracted from the IAN database questionnaire responses included initial ASD diagnosis, race, gender, birth date, initial evaluator, birth order, history of skill loss, and current address. The race variable corresponded to a category delineated by the U.S. Census Bureau and was eventually collapsed into four categories because of cell size: White, Black/African American, multiracial, and other. For the variable of intellectual disability (ID) status, participants were categorized as ID if they either (a) reported ever receiving a diagnosis of “mental retardation” or (b) reported an IQ score of <70. Exact timing of ID diagnosis in relation to ASD diagnosis is not reported. 

For history of skill loss, we reclassified skill loss as “autistic regression” if parents reported moderate to severe social or communication skills lost before age 3.

Regions were defined according to the U.S. Census Bureau, with Delaware and Maryland grouped into the Northeast [49]. Data on county urbanicity/rurality, county median income, and county racial distribution were assigned by matching Federal Information Processing Standards codes with federal data [50]. These six subgroups were later collapsed into two groups: large central or fringe metropolitan (principal city ≥1 million) and rural to medium metropolitan (metropolitan statistical area <1 million; micropolitan; rural). 

For county median income and county percentage white, categories were distributed by national quartiles.

Data on maternal education were extracted from IAN questionnaires; only 10% of biological fathers had completed forms; therefore, no paternal data were included; education data is linked to the parent filling out the form and hence, paternal education is not elicited from maternal forms (and vice versa). Maternal education history was eventually divided into two categories based on completion of bachelor's degree.

If families skipped a question based on an answer to the previous question, answered do not know, or declined to answer a question, data were recorded as missing. 

All survey data were entered by parents and maintained in the Internet Mediated Research System, IMRS (MDLogix, Baltimore, MD).

### 2.3. Statistical Analysis

Analyses were performed using STATA 11.0 (College Station, TX). Within individual analyses, we used the model-wise complete case approach to handling missing data, whereby those with missing data for a given model were not included in that analysis. 

Initial testing showed that AOC and AOD variables are not normally distributed. Therefore, both dependent outcomes were log-transformed; doing so approached a normal distribution for both AOC and AOD compared with other transformation techniques like squaring or inversion. For analysis of each independent variable's association with transformed AOC and AOD, univariate linear regression was used to identify those variables with *P* < .10 for inclusion in the multivariate model.

We then used multivariate linear regression (xtreg) with state-level clustered random effects, to examine the associations of significant covariates along with *a priori* independent variables (gender, current age, race, ethnicity, maternal education, and era of diagnosis) with the log-transformed dependent outcomes: age at first parent concern (AOC) and age at first diagnosis (AOD). The AOD model also included geographic characteristics (region, county median income quartiles, county percentage white quartiles, binary rurality) to reflect potential variation in access to diagnostic services and pediatric specialists. The random effects approach helps to correct for uncertain potential similarities clustered among certain groups; in this instance, these groups are residents of the same state. 

To interpret the results of the multivariate linear regression of log-transformed data, the coefficient was multiplied by the referent group base value, yielding difference in months between referent group and the analyzed factor, all other factors held equal. 

To assess contribution of different levels of independent variables to AOC and AOD, we assessed differences in pseudo-R-squared between each model. We then used likelihood ratio testing to test differences between nested models for AOC and AOD: base model (individual: gender, age, initial diagnosis, history of autistic regression and/or ID, high-risk (AOC only)); family characteristics (firstborn, race, ethnicity, maternal education); and geographic characteristics (region, county affluence and racial composition, rurality).

## 3. Results

Mean AOC in our registry of children of all ages (Table 1) diagnosed between 2004 and 2010 was 19.6 months (SD = 15.0), and median AOC was 18 months (interquartile range [IQR], 11.0–24.0). Mean AOD was 47.9 months (SD = 30.8) with a median AOD of 36.9 months (IQR 27.8, 56.4). Among those >8 years old, mean unadjusted AOD was 57.7 months (SD = 35.3).

As seen in Table 1, unadjusted univariate log-transformed mean AOC was later for male, African-American or Asian-American, Hispanic, and older children as well as those with initial diagnosis other than autistic disorder (AD), and firstborn status. History of intellectual disability (ID) or medical problems (extreme prematurity, cerebral palsy, and/or seizure disorder) was univariately associated with earlier AOC. Maternal education, chronological era, and geographic factors did not affect AOC. 

Mean log-transformed AOD in univariate analysis differed significantly by ethnicity, age, specific initial ASD diagnosis, birth order, and history of autistic regression; by maternal education; county percent white, rurality, region, and age category (Table 1). 

For AOC, likelihood ratio testing found significant differences in model fit with the addition of family-level characteristics (ethnicity, race, maternal education, firstborn) to individual characteristics (gender, first diagnosis, history of regression, history of intellectual disability) (LR chi-sq 43.0, *P* < .001) but not with subsequent addition of geographic factors (LR chi-sq 18.39, *P* = .073). Therefore, these geographic factors were not included in the multivariate model.

Multivariate linear regression analysis of log-transformed AOC, adjusted by age, is shown in Table 2. The referent group in the AOC analysis was white, non-Hispanic, non-firstborn males diagnosed with AD and with a history of ID and medical high-risk without regression; mean AOC for this group was 10.3 months. Parents of African-American or Hispanic children reported 1.94 and 1.56 mo later AOC than white or non-Hispanic children, respectively. Maternal education did not affect AOC. The largest risk factor for later AOC was initial diagnosis of AS (2.93 mo); the largest correlate for early AOC was high medical risk (2.8 mo earlier than reference). Children who were firstborn had 1 mo later AOC than the referent group. 

Inclusion of a gender-diagnosis interaction term was not significant in AOC.

In multivariate analysis of AOD, there were significant increases in model fit with the addition of each layer of characteristics: LR test individual with family, LR  chi^2^ = 51.03, *P* < .001; LR test individual-family with addition of geographic characteristics, LR  chi^2^ = 1930.16, *P* < .001. Adjusted *r*-squares for each model were 0.427, 0.432, and 0.582, respectively. 

In Table 3, results of log-transformed AOD multivariate linear regression with clustering for state are shown. The referent group was white male, non-Hispanic children with initial diagnosis of AD without comorbid ID, autistic regression or firstborn, with lower maternal education, living in the area with lowest white quartile, lowest income quartile, and large metropolitan area. Average AOD was 14.7 months in this calculated group.

An interaction term with gender and diagnosis in AOD was tested but was not included due to collinearity in the ultimate model.

Statistically significant risk factors for 1 mo delay in AOD included black or multiracial race (1.08, 1.45), living in neither poor nor wealthy counties (middle quartiles, 1.55–1.07 mo later), living in rural areas (.74 mo later), and living in a region other than the North East (1.5–1.7 mo later). Both ID and history of autistic regression were associated with earlier AOD. Initial diagnosis with AS was associated with over one-year later AOD than the referent group. Graduate maternal education had minimal (0.3 month earlier) effect on AOD.

## 4. Discussion

Overall, our data suggest that even among higher-resource families enrolled in an online voluntary national autism registry, individual and family factors contribute to variation in AOD and somewhat with AOC. AOD is also affected by variation in geographic characteristics. 

### 4.1. Age of Parent Concern (AOC)

We confirmed previous research on certain individual characteristics associated with earlier AOC and AOD, including history of early medical problems [26, 33] and later AOC and AOD for firstborn children [23, 25, 26, 51] and those with non-AD diagnoses [39]. Because of the wording of the AOC question, which was nonspecific to type of developmental concern, it was not surprising that those children with medical risk factors or history of ID were also more likely to have earlier AOC. 

The findings of earlier AOC for girls confirmed some previous studies showing slight but statistically significant earlier concern for girls with abnormal development [25, 28]. This slight variation may be due in part to initially earlier communication skills in girls than boys [52], such that a delay in a female is more atypical and is apparent to families and clinicians than in a male; this disparity deserves further consideration given that the model already includes diagnosis with varying degrees of gender disparity for AS versus other diagnoses.

Delays in AOC for both African-American and Hispanic children have been reported in one other published study [28]; many others, including those of a Medicaid cohort, found no difference [25, 26, 29–31]. Our findings are temporized by the overall small cell size (<3%) of underrepresented minorities; some have postulated that disparities in perception of ASD and behavior problems suggest that the way that different communities perceive and act on atypical behaviors can vary [53]. Further study of AOC in underrepresented populations with more socioeconomic diversity would have more power to elucidate true differences and establish effective interventions [54, 55].

### 4.2. Age of Diagnosis (AOD)

As expected, we found that initial diagnosis other than autistic disorder, especially AS, contributed to significantly delayed AOD, which follows the natural history of the disorder and the often milder presentation in PDD-NOS and AS [14, 33, 38, 39]. Presence of comorbid intellectual disability (ID) was associated with minimally earlier AOD, complementing previous reports finding either a lack of or inverse association between degree of ID or functional ability and AOD [33, 34]. Future studies examining variation by functional ability or degree of autism would be a better, more complete marker of role of impairment in predicting ASD diagnosis timing.

Our study suggested that gender contributes to slightly later AOD, analogous to findings by other studies [33, 36, 38]. This is contrary to findings demonstrated in a large Medicaid sample [14] and other studies [25, 26, 34, 56]. Given that girls have earlier AOC, later AOD (adjusted for AOC) suggests a confounding effect of gender which may be more linked to the well-known increased gender imbalance in diagnosed AS (~10 : 1) compared with other ASD (4 : 1) [57].

There were disparities by race in AOD as well, confirming past research in the US [32, 58] and internationally [59], but not seen in the largest surveillance study [33]. Although these findings are not individually clinically significant, viewed from the aggregate level, they suggest vulnerability even among the higher SES families of IAN. Further qualitative studies and interventions should focus on identifying issues of differential access, community and/or health care risk perception, and other barriers to timely AOD among children of color. Future quantitative studies with larger sample sizes of underrepresented minorities are needed to examine interaction between race and potential confounders.

Hispanic ethnicity (of English-speaking families), however, was not a risk factor for AOD differences, similar to the findings of the only comprehensive large, multisite epidemiologic surveillance study of ASD surveillance [33], a large Medicaid study [14], and other parent-report surveys [41, 56] although this is less consistent with studies using administrative data [32, 43, 55, 58, 60]. Because ours is a sample of convenience skewed toward a highly involved and more educated parent base, those children who remain undiagnosed are not included; selection bias is a possible cause of these findings, especially given that IAN is currently only available in English. 

We confirmed previous studies suggesting that higher maternal education was statistically associated with earlier AOD [25, 26, 30, 45], but the difference (0.3 months) is not clinically significant in a multivariate model, suggesting that other individual characteristics outweigh SES in predicting AOD, as was also reported by Shattuck et al. [33], at least among highly motivated families who participate in IAN. Further qualitative studies examining persistence and determination qualities (“advocacy”) regardless of financial or educational resources by families in obtaining a diagnosis and eventual outcomes are warranted. 

Lastly, there were expected statistically significant differences in AOD by geographic location, region [61], and county characteristics [14, 33], particularly by variation in county median income and region. While we could not approximate actual contribution to variance by these factors given software limitations, the percent change in both likelihood ratio testing and in adjusted *r*-square in the comparison of simple regression models suggests that well beyond individual and family characteristics in this higher SES sample (adj *R*-sq = 0.43), geographic factors greatly contribute to variance in AOD (adj *R*-sq = 0.58). This confirms previous reports that increased community access to diagnosis is associated with earlier AOD [33] and therefore is an identifiable area of opportunity for promoting increased awareness, screening, and services in more rural and Southern locations in the US.

### 4.3. Limitations

There are several limitations to this study. First, because this is a convenience sample, we are unable to approach true prevalence or true changes in AOD or AOC over time and any differences we report reflect only the registry population. There is some selection bias because families in IAN tend to be of higher socioeconomic status; nearly 50% have maternal education attainment of bachelor's degree or higher, while the national average for adult women is 30% [62]; however, this difference is similar to the bias in the majority of nonepidemiologic clinic-based and other survey-based studies. These same families may also be more likely to have earlier AOD overall, regardless of socioeconomic status. Nevertheless, comparisons of characteristics across IAN data are valuable in understanding differential factors influencing access to ASD care in this sample with multiple known (higher socioeconomic status and Internet usage) and unknown biases, which may lead to differential usage of health care systems.

In terms of information bias presented by an internet-based registry, we have previously discussed [63] the growing research supporting the validity of web-collected data [64]. ASD diagnoses within the online IAN registry have been clinically validated (*n* = 107) [46] and verified [47], confirming that the registry is a reliable modality for collecting clinical information. The detailed questions on all variables within IAN improve reliability; we further limited data to exclude families reporting improbable or inconsistent responses to maximize recall reliability specifically for AOC and AOD.

## 5. Conclusion

Our study suggests that multiple individual and family-level factors, as well as geographic characteristics (region and county income) affect AOD and often AOC, contributing to delay in initial diagnosis of ASD and entry into treatment, including early intervention. Two randomized controlled trials have shown that intervention for 2-year-olds that blends strategies based on principles of developmental and learning sciences results in significant language and cognitive improvements [9, 65] and social improvements [65]. 

The earliest possible timeframe for AOC and AOD in ASD is still being established; meanwhile, there is already definite variation with significant additive potential for improving timely diagnosis and subsequent receipt of services; this disparity could increase as more sophisticated screening tools emerge. Because no single factor can be identified, achieving equity in AOC, AOD and eventual intervention will require a multi-pronged approach that comprehensively addresses the smaller but cumulative cultural, educational, and health system factors which contribute to this variation. Our data can help public health officials and clinicians identify and explore modifiable disparities in AOC and AOD for potential interventions, such as for underrepresented minorities and those living in more rural or Southern states.

## Figures and Tables

**Table 1:** Comparison of mean age of first concern (AOC) and age of first diagnosis (AOD) by characteristic (*N*  =  6214); univariate testing by ANOVA on log-transformed AOC and AOD.

Characteristic	*N*	Mean age at first concern (AOC), mo. (SD)*	ANOVA *P* value/median	Mean age at first diagnosis (AOD), mo. (SD)*	ANOVA *P* value/median
Overall	6214	19.6 (15.0)		47.6 (30.8)	
Median			18 (IQR 11,24)		36.9 (IQR 27.8, 56.4)
*Gender*			< .001		.796
Male	5119	19.8		47.6	
Female	1095	18.6		49.0	
*Race *			.048		.367
White	5491	19.5		47.8	
African-American	165	21.7		49.2	
Asian/Asian-American	57	21.7		45.8	
Multiracial	244	18.7		50.0	
Other/unknown	257	19.6		46.4	
*Ethnicity*			< .05		.004
Hispanic	5709	19.9		44.0	
Not Hispanic	505	19.5		48.2	
Current age, years*			< .001		< .001
0–5	940	14.4		29.0	
6–11	3692	18.7		42.4	
12–18	1582	24.7		71.9	
*Initial ASD Diagnosis*			< .001		< .001
Autism	2535	10.5		38.3	
PDD-NOS^a^	1893	13.5		44.9	
Asperger	891	24.4		87.8	
Other ASD	895	11.9		41.7	
*Intellectual disability* ^ b^ * (ID)*			< .001		.194
Present	1229	17.8		48.3	
Absent	4971	20.0		47.8	
Firstborn	3242	20.6	< .001	49.1	< .001
Not firstborn	2971	18.5		46.5	
History of autistic regression	1511	16.9	.854	38.2	< .001
No regression	4703	20.4		51.0	
*High-risk* ^ c^			< .001		.535
Yes	650	16.0		47.4	
No	5563	20.0		47.9	
*Multiplex family*			.255		.776
Yes	872	21.0		49.4	
No	5342	19.3		47.6	
*Era of Initial Diagnosis*			.601		< .001
1994–2000	850	18.5		39.3	
2001–2008	5364	19.7		49.2	
*Maternal education*			.468		.007
≤College diploma	3098	19.5		48.9	
College+	3116	19.6		46.8	
*County median income*			.537		< .001
Lowest quartile	171	14.9		46.6	
2nd quartile	486	14.9		47.2	
3rd quartile	1211	16.3		51.1	
4th quartile	4344	14.7		49.9	
*County percent white*			.494		< .001
Lowest quartile	2206	19.3		46.6	
2nd quartile	2453	19.4		47.2	
3rd quartile	1178	20.6		51.0	
4th quartile	375	19.4		49.9	
*Rurality* ^50^			.595		< .001
Large central metro	1364	19.6		45.1	
Large fringe metro	2104	19.2		46.0	
Medium metro	1303	19.3		48.5	
Small metro	644	20.8		53.6	
Micropolitan	538	20.5		51.2	
Noncore (rural)	261	18.2		52.2	
*Binary rural*			.621		< .001
Principal city ≥ 1 million	368	19.4		45.7	
Principal city < 1 million	2746	19.8		50.6	
*US Region*			.311		< .001
Northeast	1843	18.9		44.8	
South	1808	19.4		50.1	
Midwest	1434	20.5		49.8	
West	1129	19.6		46.7	

Note -: not significant. Some totals in categories do not add up to 6214 due to missing data.

^a^PDD-NOS: Pervasive Developmental Disorder-Not Otherwise Specified.

^b^ID: Intellectual disability/mental retardation.

^c^High risk: history of prematurity (<34 weeks gestational age) and/or seizures and/or cerebral palsy; sibling with ASD not included.

**Table 2:** Multivariate linear regression of factors affecting log-transformed age at first parent concern (AOC) among 6214 individuals with ASD.

Parameter	Raw coefficient	Converted average difference in AOC compared with reference group (in mo)*	95% CI (in mo)		*P*
Female gender	−0.08	−0.78	−1.33	−0.21	.009
*Race*					
White	REF				
Black/African-American	0.17	1.94	0.36	3.77	.015
Multiracial	−0.07	−0.74	−1.77	0.45	—
Other	0.04	0.38	−0.72	1.60	—
Hispanic Ethnicity	0.14	1.56	0.58	2.63	.001
*Initial diagnosis*					
Autistic disorder	REF				
PDD-NOS^a^	0.05	0.55	−0.03	1.15	.060
Asperger Syndrome	0.25	2.93	2.00	3.95	< .001
Other ASD	−0.01	−0.08	−0.75	0.66	—
Presence of ID^b^	−0.13	−1.27	−1.79	−0.72	< .001
Firstborn	0.10	1.03	0.54	1.56	< .001
High risk^c^	−0.32	−2.82	−3.35	−2.25	< .001
History of autistic regression	0.06	0.58	0.03	1.18	.042

Adjusted by age.

Note —: *P* > .05, statistical nonsignificance.

^a^PDD-NOS: Pervasive Developmental Disorder-Not Otherwise Specified.

^b^ID: Intellectual disability/mental retardation.

^c^High risk: history of prematurity and/or seizures and/or cerebral palsy.

**Table 3:** Multivariate random-effects linear regression model (clustering by state) of log-transformed age at initial ASD diagnosis (AOD), adjusted by age and age of concern (*n* = 6214).

Parameter	Raw coefficient	Average difference in AOD compared with reference group (in mo)*	95% CI Difference (in mo)		*P*
*Gender*					
Male	REF				
Female	0.04	0.46	0.06	0.87	< .025
*Race*					
White	REF				
African-American	0.08	1.08	0.1	2.12	.031
Multiracial	0.10	1.45	0.62	2.33	< .001
Other	0.06	0.82	0.07	1.6	.032
*Ethnicity*					
Hispanic	−0.02	−.16	−0.73	0.44	—
Not Hispanic	REF				
*Initial ASD Diagnosis*					
Autism	REF				
PDD-NOS^a^	0.09	1.32	0.92	1.72	< .001
Asperger	0.62	12.39	11.49	13.32	< .001
Other ASD	0.08	1.16	0.67	1.66	< .001
*Intellectual disability* ^ b^ * (ID)*					
Present	−0.05	−0.67	−1.04	−0.28	< .001
Absent	REF		
Firstborn	0.03	0.36	0.05	0.67	.024
Not firstborn	REF				
History of autistic regression	−0.08	−1.05	−1.38	−0.72	< .001
No regression	REF				
*Maternal education*					
≤College diploma	REF				
College diploma	−0.02	−0.27	−0.42	−0.12	< .001
*County median income*					
Lowest quartile	REF				
2nd quartile	0.1	1.55	0.43	2.76	.006
3rd quartile	0.08	1.07	0.06	2.15	.038
4th quartile	0.05	0.63	−0.35	1.67	—
*County percent white*					
Lowest quartile	REF				
2nd quartile	0.02	0.20	−0.18	0.58	—
3rd quartile	0.05	0.63	0.14	1.13	.012
4th quartile	0.05	0.66	−0.08	1.43	—
*Rurality*					
*Metropolitan/peri-metro*	REF				
Large/small town/rural	0.05	0.74	0.37	1.12	< .001
*Region*					
Northeast	REF				
South	0.11	1.69	1.23	2.16	< .001
Midwest	0.11	1.65	1.18	2.13	< .001
West	0.1	1.46	0.95	1.99	< .001

Adjusted by current age and log age of concern (AOC).

Note—: *P* > .05, statistical nonsignificance.

^a^PDD-NOS: Pervasive Developmental Disorder-Not Otherwise Specified.

^b^ID: Intellectual disability/mental retardation.

^c^High risk: history of prematurity and/or seizures and/or cerebral palsy.

## Abbreviations

AOC: Age of parent first concern
AOD: Age of first diagnosis
ASD: Autism spectrum disorder
AS: Asperger syndrome/Asperger disorder
ID: Intellectual disability
IQR: Interquartile range.
